# Maternal ethnicity and the prevalence of British pregnancies affected by neural tube defects

**DOI:** 10.1002/bdr2.1893

**Published:** 2021-03-23

**Authors:** Jordana N. Peake, Rachel L. Knowles, Jill Shawe, Judith Rankin, Andrew J. Copp

**Affiliations:** 1 Developmental Biology & Cancer Department, UCL Great Ormond Street Institute of Child Health, London, UK; 2 Population, Policy & Practice Department, UCL Great Ormond Street Institute of Child Health, London, UK; 3 Faculty of Health, University of Plymouth, Devon, UK; 4 Population Health Sciences Institute, Newcastle University, Newcastle upon Tyne, UK

**Keywords:** ethnicity, neural tube defects, pregnancy, prevention, spina bifida

## Abstract

**Background:**

Few data are available on the prevalence of neural tube defects (NTDs) within different ethnic communities of the UK. This study aimed to calculate prevalence estimates for NTD-affected pregnancies, classified by maternal ethnicity, and to explore why variations in prevalence might exist.

**Methods:**

A cross-sectional study was performed with data from regional congenital anomaly registers in England and Wales, for NTD-affected pregnancies between 2006 and 2011. Using binomial regression models, we examined NTD-affected pregnancy prevalence estimates and rate ratios (PRRs), by maternal ethnicity.

**Results:**

The prevalence of NTDs was 12.14 per 10,000 births, with no differences between study years. Anencephaly, encephalocele and spina bifida occurred at 4.98, 1.37 and 5.80 per 10,000 births respectively. Mothers of Indian ethnicity were 1.84 times more likely (95% CI: 1.24, 2.73) and Bangladeshi mothers 2.86 times more likely (95% CI: 1.48, 5.53) than White mothers to have an NTD-affected pregnancy, after adjusting for maternal deprivation and maternal age. The excess prevalence in Indian mothers was specifically for anencephaly (PRR 2.57; 95% CI: 1.52, 4.34), and in Bangladeshi mothers the trend was for increased spina bifida (PRR 3.86; 95% CI: 0.72, 8.69). Anencephaly in Indian mothers was especially associated with other congenital anomalies (non-isolated NTDs).

**Conclusions:**

Different British ethnic groups vary in NTD prevalence. The excess prevalence of anencephaly as a non-isolated NTD in pregnancies of Indian mothers could indicate involvement of genetic or other unmeasured behavioral factors. Future work is needed to seek etiological explanations for the ethnicity differences and to develop improved methods for primary prevention.

## Introduction

Neural tube defects (NTDs) are severe congenital anomalies resulting from failed closure or subsequent abnormal development of the embryonic neural tube. Closure is normally complete by day 28 of pregnancy, before many women know they are pregnant (Molloy, Pangilinan, & Brody, 2017). NTDs affect an average of 1 in 1000 pregnancies worldwide, although wide variations in prevalence exist, depending on the population under study (Copp, Stanier, & Greene, 2013; Zaganjor et al., 2016; Molloy et al., 2017). The most common sub-types are anencephaly and open spina bifida (myelomeningocele), and NTDs can occur in isolation or in association with other congenital anomalies (Frey & Hauser, 2003; Mitchell, 2005). Termination of pregnancy for fetal anomaly (TOPFA) was the outcome for 81% of NTD-affected pregnancies in the UK between 1991 and 2012 (Morris et al., 2016).

NTDs have multifactorial causation, in which a genetic predisposition interacts with non-genetic influences (Carter, 1974; Au, Ashley-Koch, & Northrup, 2010; Copp et al., 2013). Factors shown to be associated with NTD risk include socio-economic status, maternal age, maternal ethnicity, maternal diabetes, maternal obesity, parental occupational exposures, hyperthermia during early pregnancy, previous spontaneous abortions, maternal use of valproic acid and fetal gender (Frey & Hauser, 2003; Au et al., 2010; Copp et al., 2013; Tanoshima et al., 2015).

A significant finding to emerge from NTD epidemiological studies is the link between folate intake before and during early pregnancy (peri-conceptionally) and NTD occurrence and recurrence. The 1991 Medical Research Council multicenter randomized controlled trial (RCT) demonstrated a 72% reduction in NTD recurrence in women taking folic acid supplements (MRC Vitamin Study Research Group, 1991) and a second RCT confirmed that folic acid, when taken together with other vitamins, also has a protective effect for first occurrence NTDs (Czeizel & Dudás, 1992). A large-scale population study in China demonstrated a significant reduction in the historically very high NTD prevalence following introduction of folic acid supplementation (Berry et al., 1999). From 1998, the USA and subsequently many other countries have adopted mandatory fortification of bread flour with folic acid (Castillo-Lancellotti, Tur, & Uauy, 2013; Osterhues, Ali, & Michels, 2013). Fortification has not been implemented in the UK, or in any other European country, and this seems likely to have been a missed opportunity for enhanced primary prevention of NTDs (Morris et al., 2016). Indeed, across Europe between 1991 and 2011 there was no clear evidence of a downward trend in NTD prevalence (Khoshnood et al., 2015).

Our systematic review of the literature found that women of White ethnicity are more likely to take folic acid before pregnancy than those of non-White ethnicity (Peake, Copp, & Shawe, 2013). Nevertheless, there is a paucity of data on NTD prevalence by maternal ethnicity in the UK. Preliminary findings suggest that the rate of NTD-affected pregnancies (including TOPFAs) is higher in women of Pakistani and Indian origin (Tonks, Wyldes, & Whittle, 1995; Michie, Chambers, Abramsky, & Kooner, 1998), and that stillbirth and infant mortality rates attributed to NTDs are high in the Bangladeshi community (Balarajan & McDowall, 1985). Higher maternal age and consanguinity, where mother and father are close blood relations, appear to contribute to higher NTD prevalence for mothers of Pakistani ethnicity but not for mothers of Indian ethnicity (Terry, Bissenden, Condie, & Mathew, 1985; Young & Clarke, 1987; Chitty & Winter, 1989; Sheridan et al., 2013).

We aimed to calculate NTD prevalence estimates for British mothers from different ethnic groups, and to further examine why variations with maternal ethnicity might exist. Critically, we used a data source that has high ascertainment of data from TOPFAs, as well as live and still births. We took into account both NTD type and whether NTDs are isolated or associated with other congenital anomalies, and examined the importance of maternal age and deprivation, which have largely been under-represented in previous studies.

## Methods

### Data sources

Until 2015, the British Isles Network of Congenital Anomaly Registers (BINOCAR), a network of regional congenital anomaly registers across the UK and Ireland, was the primary source of population-based congenital anomaly data (Boyd et al., 2005). BINOCAR received data from multiple sources, including cytogenetic and post-mortem reports and prenatal diagnosis (Boyd et al., 2005), ensuring high levels of ascertainment as confirmed through comparisons with the European Surveillance of Congenital Anomalies (EUROCAT) (Rankin et al., 2005).

Pseudonymised, individual-level data were obtained for all NTD cases notified between 01 Jan 2006 and 31 Dec 2011 to five BINOCAR registers: the East Midlands and South Yorkshire Congenital Anomalies Register (EMSYCAR), the Northern Congenital Abnormality Survey (NorCAS), South West Congenital Anomaly Register (SWCAR), the Congenital Anomaly Register for Oxfordshire, Berkshire and Buckinghamshire (CAROBB) and the Congenital Anomaly Register and Information Service for Wales (CARIS). These specific registers were included as they all collected information on self-reported maternal ethnicity, which was adapted from the Office for National Statistics (ONS) 2001 census classification (Office for National Statistics, 2019). Based on the data obtained from the registers, maternal ethnicity was thus categorized as White, Indian, Pakistani, Bangladeshi, Black Caribbean, Black African or Other (which included Mixed, Other Asian, Other Black and Chinese). Deprivation quintile was collected by all registers, based on index of multiple deprivation (IMD) scores of maternal residence (Communities and Local Government, 2011; Welsh Government, 2011) at pregnancy outcome, with quintile 5 being the least deprived and quintile 1 the most deprived. Maternal age in absolute years was collected by all registers and grouped into different age ranges (<20; 20-24; 25-29; 30-34; 35-39; 40+) for analysis. All mothers aged 16 years and over were included. Gender of fetus, maternal body mass index (BMI) and folic acid usage data were also initially obtained but could not be included in analyses due to the sparsity of available data.

NTDs were classified by sub-type (anencephaly, encephalocele, spina bifida) and whether they were ‘isolated’ or ‘non-isolated’. All BINOCAR registers used a multiple malformation group variable, generated using EUROCAT’s multiple congenital anomaly algorithm. This assigns congenital anomalies to categories based on International Classification of Diseases (ICD) 10 coding. This indicated whether an NTD occurred in isolation or was non-isolated: i.e. with at least one other associated anomaly or as part of a recognized syndrome. For the ethnicity analysis, however, only data from EMSYCAR and SWCAR were used (see below) and, given the relatively small NTD case numbers in this data subset, a more precise method of assigning NTDs to isolated/non-isolated categories was applied. EMSYCAR collected a local etiological classification variable, which provides a more accurate representation of whether an NTD is isolated or not, as each case is reviewed individually and coded accordingly. Hence, the isolated/non-isolated distinction within the ethnicity analysis was based on this local etiological classification variable, using data only from EMSYCAR.

Live and stillbirth denominator data were obtained from the ONS. Approximately 30% of births to mothers in England and Wales, during the timeframe of the study, occurred in the regions covered by the five BINOCAR registers. The distribution of births by maternal age and maternal ethnicity across the registers is reflective of England and Wales as a whole. TOPFA data were obtained from the Department of Health, covering the same regions as the registers.

### NTD prevalence calculation and data stratification

NTD prevalence was obtained from the BINOCAR birth prevalence calculation. This has the number of congenital anomaly cases resulting in live births (CA_LB_), stillbirths (CA_SB_), late miscarriages (> 20 weeks gestation)(CA_LM_) and TOPFA (CA_TOPFA_) in the numerator and the total number of live births (LB) and stillbirths (SB) in the population in the denominator:

$$p=10,000\times \frac{CA_{LB}+CA_{SB}+CA_{LM}+CA_{TOPFA}}{LB+SB}$$

However, prevalence was calculated for NTD-affected pregnancies rather than individual NTD cases to ensure that maternal ethnicity would not be counted twice for the same pregnancy, i.e. in twin pregnancies where both fetuses had been registered as having an NTD. Although for NTD-affected pregnancy prevalence calculations, ethnicity of the mother is in the numerator (BINOCAR data) and ethnicity of the baby in the denominator (ONS data), strong agreement between the two has previously been demonstrated (Dattani, Datta-Nemdharry, & Macfarlane, 2011, 2012).

### Ethnicity data analysis

Over 30% of ethnicity data for NorCAS, CAROBB and CARIS were missing (46%, 38% and 32%, respectively) which precluded use of these registries for ethnicity analysis, as ignoring missing data was shown to introduce bias and imprecision. As ethnicity data were not missing at random, they could not be imputed. Ethnicity data were missing in only 13% and 7% respectively in EMSYCAR and SWCAR, and so analyses of ethnicity effects were conducted using only data from these registries. Univariable explorations of the association between NTD prevalence, maternal age, maternal deprivation and maternal ethnicity were first conducted and then potentially confounding factors: maternal deprivation and maternal age, were added iteratively into a binomial regression model exploring the association between maternal ethnicity and NTD-affected pregnancy prevalence. Data were stratified by NTD sub-type although, due to the small number of encephalocele-affected pregnancies, these were included in analyses of total NTDs, but excluded from sub-type analyses. Data were also stratified by whether or not the NTD was isolated (discussed above). Finally, sensitivity analyses were conducted to explore the impact on the model of removing NTD-affected pregnancies that occurred as part of a multiple set. Stata versions 12 and 13 (StataCorp LLC, USA) were used to clean and analyse the data.

### Sample size calculation

Using published evidence of expected effect sizes (Balarajan & McDowall, 1985; Chitty & Winter, 1990; Tonks et al., 1995), a sample size estimate was performed to calculate the minimum number of mothers from different ethnic groups required in the analysis to detect NTD rate differences.

### Regulatory approvals

NHS Research Ethics Committee (reference: 12/LO/0890) and section 251 approval (reference: ECC 5-05(d)/2012) were obtained.

## Results

**Figure 1:**
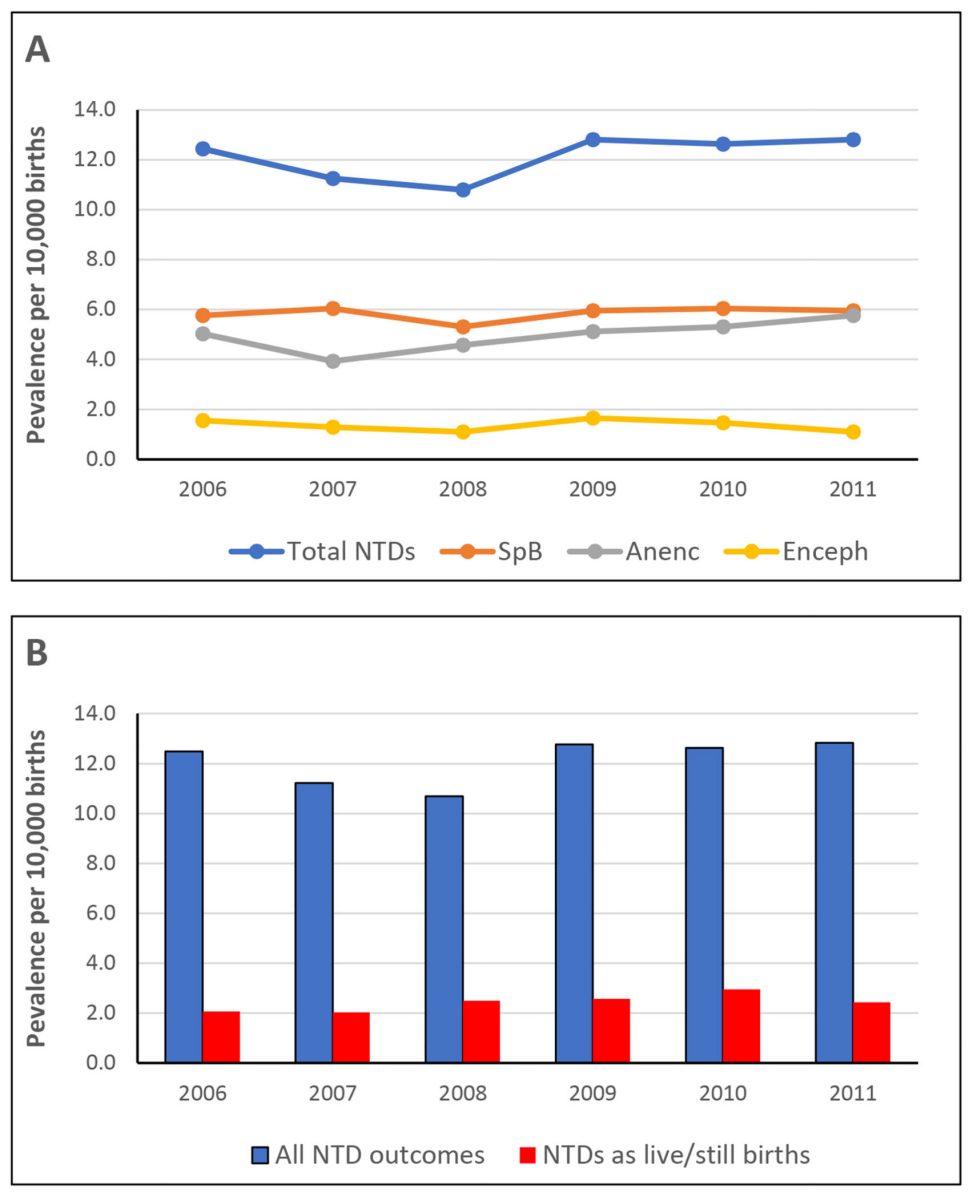
Prevalence of NTDs per 10,000 births in England and Wales by year, 2006-2011. (**A**) Prevalence of total NTDs (blue) and the main NTD sub-types: spina bifida (SpB, brown), anencephaly (Anenc, grey) and encephalocele (Enceph, yellow). (**B**) Prevalence of NTD outcomes, shown as (in blue) the combined numbers of NTDs in live births (LB), stillbirths (SB), terminations of pregnancy for fetal anomaly (TOPFAs) and late miscarriages, or (in red) as LB and SB only. There are no significant differences between years in prevalence of any NTD sub-type or NTD outcome.

There were no statistically significant differences in NTD prevalence by year in the pooled data from the five registers (Figure 1A). As a result, data for all years were combined for subsequent analyses of absolute prevalence and prevalence rate ratios (PRRs). Fewer than 20% of NTDs presented as LB or SB in the pooled register data, with the remainder, around 80%, presenting mainly as TOPFAs. There were no significant differences in PRRs from year to year (Figure 1B).

The combined data for 2006-2011, from the five congenital malformation registers: EMSYCAR, NorCAS, SWCAR, CAROBB and CARIS, give an overall NTD prevalence of 12.14 per 10,000 births. Anencephaly, encephalocele and spina bifida were present at 4.98, 1.37 and 5.80 per 10,000 births, respectively (Table 1). Overall, approximately three quarters of the NTDs were isolated (with no other major co-existing malformations) while the remainder were non-isolated.

Table 2 shows PRRs by maternal age group and by index of multiple deprivation (IMD). For maternal age, the 25-29 age group was selected as reference group due to the highest numbers of births being in this category. The overall NTD prevalence is significantly higher in the < 20 group (p = 0.028) and significantly lower in the 30-34 age group (p = 0.036), compared with the 25-29 reference group. No statistically significant differences were detected by NTD sub-type.

For IMD, quintile 1 (most deprived) was selected as reference group due to the highest numbers of births being in this category. Overall, NTD prevalence is significantly lower in quintiles 4 and 5 than quintile 1. This apparent trend was tested by fitting IMD quintile as a continuous variable, which showed that for each unit increase of quintile, the risk ratio was 0.93 (95% CI: 0.90, 0.96) (p<0.001). Hence, NTD prevalence is higher in more deprived areas. When considering anencephaly and spina bifida individually, a similar outcome is observed for quintile 4, but not for quintile 5 where the difference did not reach statistical significance (p = 0.06).

Ethnicity effects on NTD prevalence were examined using combined data from EMSYCAR and SWCAR, as these were the only registers with > 85% completeness of ethnicity recording. There were 748 NTD-affected pregnancies, with a denominator of 651,303 births, giving an overall NTD-affected pregnancy prevalence of 11.48 per 10,000 births. NTD prevalence was significantly higher for mothers of Indian and Bangladeshi ethnicity (21.38 per 10,000 births [95% CI: 14.55, 31.42] and 35.66 per 10,000 births [95% CI: 18.53, 68.61], respectively), compared with mothers of White ethnicity (11.49 per 10,000 births [95% CI: 10.65, 12.41]) (Table 3). Mothers of Indian ethnicity had a particularly marked prevalence of anencephaly-affected pregnancies, whereas Bangladeshi mothers showed a high prevalence of spina bifida-affected pregnancies (Table 3).

Adjusting for maternal deprivation and maternal age was found to have little impact on the observed association between ethnicity and NTD-affected pregnancy prevalence in the binomial regression model (Table 4). Specifically, in the adjusted model, mothers of Indian ethnicity were still 1.84 times more likely (95% CI: 1.24, 2.73) and Bangladeshi mothers 2.86 more likely (95% CI: 1.48, 5.53) than White mothers to have an NTD-affected pregnancy (Table 4).

When stratifying by NTD sub-type (anencephaly or spina bifida) and adjusting for maternal deprivation and maternal age, the anencephaly prevalence was still 2.57 times higher for Indian mothers (95% CI: 1.52, 4.34) and the spina bifida prevalence remained 3.86 times higher for Bangladeshi mothers, compared with White mothers (95% CI: 0.72, 8.69) (Appendix 1).

When stratifying by whether the NTD was isolated or non-isolated, and by NTD sub-type (using only EMSYCAR data; Appendices 2 and 3), the prevalence excess for anencephaly-affected pregnancies for Indian mothers compared with White mothers was more marked for the non-isolated (PRR 7.52; 95% CI: 2.82, 20.09; Appendix 3) than for isolated NTDs (PRR 2.44; 95% CI: 1.23, 4.81; Appendix 2). EMSYCAR had small numbers of NTDs in Bangladeshi pregnancies, therefore precluding definite conclusions on the preponderance of spina bifida in isolated cases.

In sensitivity analyses, removal of the 35 NTD-affected pregnancies that occurred as part of a multiple set (with only one individual within each set affected by an NTD) across the two congenital anomaly registers (EMSYCAR and SWCAR) was found to have very little impact on the results (Appendix 4: compare with Table 4).

## Discussion

This study presents estimates of NTD prevalence by maternal ethnicity using data from England and Wales. Importantly, pregnancy terminations due to fetal anomaly are included, as these make up a large proportion of NTD cases. We find that mothers of Indian and Bangladeshi ethnicity have a significantly higher NTD prevalence than mothers of White ethnicity. The excess prevalence in Indian mothers is particularly for anencephaly-affected pregnancies and in Bangladeshi mothers for spina bifida-affected pregnancies. There are also indications that the prevalence excess for Indian mothers is more marked for non-isolated than isolated anencephaly-affected pregnancies. Adjusting for maternal deprivation and maternal age had little impact on any of the observed NTD prevalence discrepancies by maternal ethnicity.

Our findings for the English regions covered by the EMSYCAR and SWCAR (East Midlands, South Yorkshire and Southwest England) are consistent with previous research conducted in the UK which indicated a higher NTD prevalence in mothers of Indian ethnicity (Balarajan & McDowall, 1985; Terry et al., 1985; Michie et al., 1998). In studies undertaken in India, a high NTD prevalence has also been observed, particularly in the North but also elsewhere (Verma, 1978; Allagh et al., 2015; Bhide, Gund, & Kar, 2016; Cherian et al., 2016). In line with our findings, one of these studies reported a high rate of anencephaly-affected pregnancies specifically (Verma, 1978) and a further study identified a high rate of anencephaly-affected pregnancies occurring in association with other anomalies (Gole, Meshram, & Hattangdi, 2014). Isolated NTDs (that lack co-existing anomalies in other body systems) are considered etiologically distinct from non-isolated NTDs (where other congenital anomalies are also present), even when NTDs occurring as part of known chromosomal, genetic or teratogenic syndromes are excluded (Frey & Hauser, 2003). It is argued that non-isolated NTDs are less likely than isolated ones to decline in prevalence with folic acid usage (Stevenson, Seaver, Collins, & Dean, 2004; Stoll, Dott, Alembik, & Roth, 2011). Mouse studies have shown that mutation of a single gene, which is necessary for correct development of more than one body system, can result in non-isolated NTDs (Greene, Massa, & Copp, 2009). Thus, the preponderance of non-isolated anencephaly in mothers of Indian ethnicity may indicate the particular involvement of genetic factors (Frey & Hauser, 2003). Published literature on NTD prevalence within Bangladeshi mothers is more limited. Only one study has described an excess infant mortality due to NTDs in Bangladeshi and Indian mothers in the UK (Balarajan & McDowall, 1985). Nevertheless, the World Health Organization has reported that Bangladesh is one of the countries in South East Asia with a high NTD prevalence (World Health Organisation, 2013). An NTD excess was not observed for Pakistani mothers in the current study, despite an increased NTD prevalence being previously observed in mothers of this ethnic group in other UK regions (Chitty & Winter, 1990; Tonks et al., 1995; Michie et al., 1998; Sheridan et al., 2013). Such a discrepancy could be due to true regional differences or a reflection of small sample sizes.

The addition of maternal age to any of the regression models was shown to have little impact on the observed association between ethnicity and NTD prevalence. This supports previous findings that maternal age is not a significant factor in congenital anomaly risk for Indian mothers (Terry et al., 1985). We also stratified by maternal deprivation - a key novel aspect of the current research - but this was shown to have little impact on any observed NTD prevalence discrepancies by maternal ethnicity. Hence, ethnic differences in NTD prevalence cannot be explained solely by socio-economic factors.

### Study limitations

Analysis of NTD prevalence in relation to ethnicity was conducted using only EMSYCAR and SWCAR data, due to the high proportion of missing ethnicity data in other BINOCAR registers. However, the statistically significant NTD excess we observed in Indian mothers is in agreement with previous findings on the epidemiology of NTDs in Indian mothers. In contrast, the observed excess of spina bifida in Bangladeshi mothers was more unexpected (based on the literature). While this finding is based on a relatively small number of NTDs, nevertheless, it would be important to explore whether an increase of spina bifida among Bangladeshi mothers is also evident in other geographical areas.

Some important potential risk factors could not be explored using the available data, including maternal body mass index and prevalence of diabetes, occupation, hyperthermia exposure, previous abortions, and valproic acid usage. Dietary habits such as vegetarianism and, critically, folic acid usage are important factors, but the BINOCAR data were too incomplete for these variables to allow analysis. Maternal deprivation shows a strong negative correlation with folic acid usage (Brough, Rees, Crawford, & Dorman, 2009) suggesting it may be used as a proxy measure. Although it was not possible to explore the influence of religion or consanguinity in the current study, the majority of Indian mothers with an NTD-affected pregnancy in this study came from Leicester, where the largest non-Christian religious group is Hindu (Leicester City Council, 2006). It has been shown that consanguinity is rare in Sikh and Hindu mothers (Young & Clarke, 1987). Consanguinity could have influenced the risk for mothers of Bangladeshi ethnicity as it is likely to be a factor in populations that are predominantly Muslim, with significant numbers of first-cousin marriages (Bittles & Black, 2010).

### Conclusions

Our finding of an enhanced anencephaly prevalence among Indian mothers, compared with White mothers, after adjustment for maternal age and deprivation, and particularly in non-isolated rather than isolated NTD cases, may suggest the involvement of genetic factors. The persistently high NTD prevalence in Indian mothers, regardless of where they reside, also points to genetic factors. There is a need to better characterize these non-isolated NTDs in Indian mothers, including genomic studies to explore genetic causation. There is also a clear excess of spina bifida-affected pregnancies in Bangladeshi mothers for the regions studied but there may be geographical variation in NTD prevalence for mothers of this ethnic group. NTD rates in mothers of Pakistani ethnicity remain unclear, due to discrepancies between previous findings and the current study.

## Supplementary Material

Appendices

## Figures and Tables

**Table 1:** Prevalence of NTD-affected pregnancies per 10,000 births (95% CI), by NTD type and whether the NTD was isolated or non-isolated^1^. From combined EMSYCAR, NorCAS, SWCAR, CAROBB and CARIS data.

NTD type	Anencephaly (n = 673)	Encephalocele (n = 185)	Spina bifida (n = 784)	Total NTDs (n = 1642)
Isolated (n = 1248)	4.14 (3.81-4.50)	0.79 (0.65-0.96)	4.30 (3.95-4.66)	9.23 (8.73-9.76)
Non-isolated (n = 394)	0.84 (0.69-1.01)	0.57 (0.46-0.72)	1.50 (1.30-1.72)	2.91 (2.63-3.22)
All NTDs	4.98 (4.61-5.37)	1.37 (1.18-1.58)	5.80 (5.40-6.22)	12.14 (11.56-12.75)

1 Determined using the multiple malformation group variable.

**Table 2:** NTD-affected pregnancy prevalence per 10,000 births and prevalence rate ratios (95% CI) for anencephaly and spina bifida, by maternal age group^1^ and maternal deprivation^2^.

		Anencephaly		Spina Bifida		Total NTDs	
	No. NTD-affected pregnancies	Prevalence per 10,000 births	Prevalence rate ratio (PRR)	Prevalence per 10,000 births	Prevalence rate ratio (PRR)	Prevalence per 10,000 births	Prevalence rate ratio
**Maternal age**							
<20	142	6.48(4.96-8.32)	1.33(0.99-1.77)	7.33(5.70-9.27)	1.26(0.96-1.66)	15.08(12.71-17.77)	1.24(1.02-1.49)^3^
20-24	358	5.61(4.76-6.57)	1.15(0.93-1.43)	6.02(5.13-7.01)	1.04(0.85-1.27)	13.05(11.73-14.47)	1.07(0.93-1.23)
**25-29 (Ref)**	**450**	**4.88(4.19-5.65)**	**1.0**	**5.80(5.05-6.64)**	**1.0**	**12.20(11.10-13.38)**	**1.0**
30-34	380	4.19(3.55-4.92)	0.86(0.69-1.07)	5.19(4.47-5.99)	0.89(0.74-1.09)	10.55(9.52-11.66)	0.86(0.75-0.99)^4^
35-39	247	4.77(3.88-5.81)	0.98(0.77-1.25)	5.88(4.89-7.02)	1.01(0.81-1.27)	11.91(10.47-13.49)	0.98(0.84-1.14)
40+	57	5.62(3.67-8.23)	1.15(0.76-1.74)	4.75(2.98-7.19)	0.82(0.53-1.27)	12.31(9.33-15.95)	1.01(0.77-1.33)
**Deprivation quintile (IMD)**							
**1 (most deprived)(Ref)**	**352**	**5.58 (5.01-6.19)**	**1.0**	**6.47 (5.81-7.18)**	**1.0**	**13.09 (11.76-14.53)**	**1.0**
2	283	5.19 (4.60-5.83)	0.93(0.73-1.17)	5.31 (4.71-5.96)	0.82(0.65-1.03)	11.78 (10.45-13.24)	0.90(0.77-1.05)
3	246	4.86 (4.27-5.50)	0.87(0.68-1.12)	5.11 (4.49-5.79)	0.79(0.62-1.01)	11.39 (10.01-12.90)	0.87(0.74-1.02)
4	200	4.02 (3.48-4.62)	0.72(0.55-0.94)^5^	4.66 (4.04-5.35)	0.72(0.56-0.92)^5^	9.95 (8.62-11.43)	0.76(0.64-0.91)^5^
5 (least deprived)	224	4.35 (3.80-4.96)	0.78(0.61-1.01)	5.18(4.52-5.90)	0.80(0.63-1.01)	10.47 (9.15-11.94)	0.80(0.67-0.94)^5^

1 For all registers: EMSYCAR, NorCAS, SWCAR, CAROBB and CARIS.

2 For EMSYCAR, NorCAS, SWCAR and CAROBB only, as English and Welsh IMDs are not directly comparable.

3 Significantly higher than the reference (25-29) age group, p = 0.028.

4 Significantly lower than the reference (25-29) age group, p = 0.036.

5 Significantly lower than the reference (quintile 1) deprivation group, p < 0.05.

**Table 3:** NTD-affected pregnancy prevalence estimates per 10,000 births (95% CI), by maternal ethnicity^1^.

	Total no. NTD-affected pregnancies	Prevalence per 10,000 births (95% CI)		
		Anencephaly	Spina bifida	Total NTDs
White	655	4.80 (4.27-5.41)	5.52 (4.95-6.17)	11.49 (10.65-12.41)
Indian	26	12.33 (7.43-20.45)	5.75 (2.74-12.06)	21.38 (14.55-31.42)
Pakistani	17	6.16 (2.93-12.92)	6.16 (2.93-12.92)	14.96 (9.30-24.08)
Bangladeshi	9	7.90 (1.98-31.61)	23.74 (10.66-52.90)	35.66 (18.53-68.61)
Black Caribbean	5	2.88 (0.41-20.43)	11.52 (4.32-30.71)	14.41 (5.99-34.63)
Black African	14	5.78 (2.60-12.87)	5.78 (2.60-12.87)	13.50 (7.99-22.80)
Other	22	2.45 (1.32-4.56)	2.21 (1.15-4.24)	5.40 (3.55-8.20)

1 Data from EMSYCAR and SWCAR.

**Table 4:** Binomial regression model to explore the association between maternal ethnicity and NTD-affected pregnancy prevalence, unadjusted and adjusted for maternal deprivation and maternal age^1^.

Variable	Unadjusted PRR	95% CI	P-value	Adjusted PRR	95% CI	P-value
**White (ref)**			0.019			0.003
Indian	1.86	1.26-2.75		1.84	1.24-2.73	
Pakistani	1.3	0.80-2.11		1.12	0.68-1.85	
Bangladeshi	3.1	1.61-5.97		2.86	1.48-5.53	
Black Caribbean	1.25	0.52-3.02		1.1	0.46-2.66	
Black African	1.17	0.69-1.99		1.04	0.61-1.77	
Other ethnic group	0.47	0.31-0.72		0.42	0.27-0.66	
**IMD quintile 1 (ref) (most deprived)**			<0.001			<0.001
IMD quintile 2	0.87	0.72-1.05		0.87	0.71-1.06	
IMD quintile 3	0.74	0.60-0.91		0.7	0.56-0.87	
IMD quintile 4	0.67	0.54-0.83		0.68	0.53-0.85	
IMD quintile 5 (least deprived)	0.7	0.56-0.87		0.69	0.54-0.88	
**25-29 (ref)**			0.378			0.367
<20	1.32	1.02-1.72		1.31	0.99-1.74	
20-24	1.03	0.84-1.26		1.01	0.81-1.25	
30-34	0.94	0.78-1.14		1.04	0.85-1.28	
35-39	1.07	0.87-1.33		1.23	0.98-1.55	
40+	1.14	0.78-1.65		1.32	0.90-1.94	

1 Data from EMSYCAR and SWCAR.
