# Supplementary material for: Maternal ethnicity and the prevalence of British pregnancies affected by neural tube defects
Source: Birth Defects Res. Author manuscript; Available in PMC 2021 Aug 27. (PMC7611580; doi:10.1002/bdr2.1893)
Supplement: Appendices [file EMS133327-supplement-Appendices.docx]

**APPENDICES**

**Appendix 1:** Binomial regression model to explore the association between maternal ethnicity and NTD-affected pregnancy prevalence, stratified by NTD type, and adjusted for maternal deprivation and maternal age. Data from EMSYCAR and SWCAR.

| **Variable** | **Anencephaly** | | | **Spina bifida** | | |
| --- | --- | --- | --- | --- | --- | --- |
|  | **Adjusted PRR** | **95% CI** | **P-value** | **Adjusted PRR** | **95% CI** | **P-value** |
| **White (ref)** |  |  | 0.037 |  |  | 0.023 |
| Indian | 2.57 | 1.52-4.34 |  | 1.02 | 0.48-2.16 |  |
| Pakistani | 1.00 | 0.44-2.26 |  | 0.98 | 0.46-2.09 |  |
| Bangladeshi | 1.50 | 0.37-6.04 |  | 3.86 | 0.72-8.69 |  |
| Black Caribbean | 0.51 | 0.07-3.63 |  | 1.79 | 0.66-4.81 |  |
| Black African | 1.06 | 0.47-2.41 |  | 0.87 | 0.38-1.96 |  |
| Other ethnic group | 0.43 | 0.22-0.84 |  | 0.37 | 0.19-0.72 |  |
| **IMD quintile 1 (ref)** |  |  | 0.001 |  |  | 0.001 |
| IMD quintile 2 | 0.93 | 0.68-1.26 |  | 0.70 | 0.52-0.94 |  |
| IMD quintile 3 | 0.72 | 0.51-1.01 |  | 0.58 | 0.42-0.81 |  |
| IMD quintile 4 | 0.61 | 0.42-0.88 |  | 0.61 | 0.44-0.85 |  |
| IMD quintile 5 | 0.66 | 0.45-0.97 |  | 0.63 | 0.45-0.89 |  |
| **25-29 (ref)** |  |  | 0.604 |  |  | 0.383 |
| <20 | 1.34 | 0.86-2.09 |  | 1.37 | 0.91-2.05 |  |
| 20-24 | 1.20 | 0.87-1.66 |  | 0.95 | 0.69-1.31 |  |
| 30-34 | 1.02 | 0.74-1.41 |  | 1.15 | 0.86-1.55 |  |
| 35-39 | 1.19 | 0.83-1.72 |  | 1.42 | 1.03-1.97 |  |
| 40+ | 2.02 | 1.20-3.38 |  | 0.86 | 0.43-1.72 |  |

**Appendix 2:** Binomial regression model to explore the association between maternal ethnicity and NTD-affected pregnancy prevalence for isolated NTDs, stratified by whether NTD type and adjusted for maternal deprivation and maternal age. Data from EMSYCAR.

| **Variable** | **Anencephaly** | | | **Spina bifida** | | |
| --- | --- | --- | --- | --- | --- | --- |
|  | **Adjusted PRR** | **95% CI** | **P-value** | **Adjusted PRR** | **95% CI** | **P-value** |
| **White (ref)** |  |  | 0.163 |  |  | 0.054 |
| Indian | 2.44 | 1.23-4.81 |  | 0.91 | 0.34-2.47 |  |
| Pakistani | 0.69 | 0.22-2.18 |  | 1.11 | 0.49-2.54 |  |
| Bangladeshi | 0.00 |  |  | 4.12 | 1.52-11.19 |  |
| Black Caribbean | 0.78 | 0.11-5.62 |  | 1.32 | 0.33-5.34 |  |
| Black African | 0.63 | 0.16-2.57 |  | 0.99 | 0.36-2.71 |  |
| Other ethnic group | 0.61 | 0.28-1.30 |  | 0.29 | 0.11-0.79 |  |
| **IMD quintile 1 (ref)** |  |  | 0.166 |  |  | 0.002 |
| IMD quintile 2 | 0.97 | 0.64-1.47 |  | 0.81 | 0.55-1.20 |  |
| IMD quintile 3 | 0.66 | 0.40-1.11 |  | 0.46 | 0.27-0.77 |  |
| IMD quintile 4 | 0.73 | 0.44-1.24 |  | 0.64 | 0.40-1.04 |  |
| IMD quintile 5 | 0.78 | 0.46-1.31 |  | 0.54 | 0.32-0.91 |  |
| **25-29 (ref)** |  |  | 0.965 |  |  | 0.664 |
| <20 | 2.07 | 1.17-3.66 |  | 1.65 | 0.95-2.87 |  |
| 20-24 | 1.33 | 0.82-2.15 |  | 1.19 | 0.76-1.85 |  |
| 30-34 | 1.25 | 0.78-2.01 |  | 1.41 | 0.92-2.17 |  |
| 35-39 | 1.86 | 1.12-3.08 |  | 1.59 | 0.97-2.60 |  |
| 40+ | 1.15 | 0.41-3.26 |  | 1.02 | 0.36-2.86 |  |

**Appendix 3:** Binomial regression model to explore the association between maternal ethnicity and NTD-affected pregnancy prevalence for non-isolated NTDs, stratified by whether the NTD is anencephaly or spina bifida and adjusted for maternal deprivation and maternal age. Data from EMSYCAR.

| **Variable** | **Anencephaly** | | | **Spina bifida** | | |
| --- | --- | --- | --- | --- | --- | --- |
|  | **Adjusted PRR** | **95% CI** | **P-value** | **Adjusted PRR** | **95% CI** | **P-value** |
| **White (ref)** |  |  | 0.980 |  |  | 0.382 |
| Indian | 7.52 | 2.82-20.09 |  | 1.06 | 0.14-7.80 |  |
| Pakistani | 2.74 | 0.63-11.83 |  | 0.98 | 0.13-7.34 |  |
| Bangladeshi | 7.79 | 1.03-58.60 |  | 5.70 | 0.76-42.57 |  |
| Black Caribbean | 0.00 |  |  | 3.85 | 0.52-28.68 |  |
| Black African | 1.91 | 0.25-14.57 |  | 1.33 | 0.18-10.00 |  |
| Other ethnic group | 0.51 | 0.07-3.77 |  | 0.00 |  |  |
| **IMD quintile 1 (ref)** |  |  | 0.186 |  |  | 0.815 |
| IMD quintile 2 | 1.69 | 0.69-4.13 |  | 0.98 | 0.39-2.47 |  |
| IMD quintile 3 | 1.25 | 0.43-3.59 |  | 0.44 | 0.12-1.59 |  |
| IMD quintile 4 | 1.15 | 0.37-3.59 |  | 0.93 | 0.33-2.64 |  |
| IMD quintile 5 | 0.47 | 0.10-2.24 |  | 1.09 | 0.40-2.99 |  |
| **25-29 (ref)** |  |  | 0.102 |  |  | 0.105 |
| <20 | 0.94 | 0.20-4.39 |  | 0.36 | 0.05-2.87 |  |
| 20-24 | 0.97 | 0.37-2.58 |  | 0.75 | 0.27-2.08 |  |
| 30-34 | 0.59 | 0.20-1.73 |  | 0.99 | 0.40-2.47 |  |
| 35-39 | 0.70 | 0.19-2.57 |  | 1.40 | 0.53-3.73 |  |
| 40+ | 6.45 | 2.32-17.93 |  | 1.78 | 0.39-8.20 |  |

**Appendix 4:** Binomial regression model to explore the association between maternal ethnicity and NTD-affected pregnancy prevalence, adjusted for maternal deprivation and maternal age, with multiples removed. Data from EMSYCAR and SWCAR.

| **Variable** | **Adjusted PRR** | **95% CI** | **P-value** |
| --- | --- | --- | --- |
| **White (ref)** |  |  | 0.009 |
| Indian | 1.87 | 1.25-2.79 |  |
| Pakistani | 1.19 | 0.72-1.96 |  |
| Bangladeshi | 3.02 | 1.56-5.84 |  |
| Black Caribbean | 1.17 | 0.48-2.82 |  |
| Black African | 1.10 | 0.65-1.88 |  |
| Other ethnic group | 0.45 | 0.29-0.69 |  |
| **IMD quintile 1 (ref)** |  |  | 0.000 |
| IMD quintile 2 | 0.88 | 0.72-1.09 |  |
| IMD quintile 3 | 0.72 | 0.57-0.90 |  |
| IMD quintile 4 | 0.71 | 0.56-0.90 |  |
| IMD quintile 5 | 0.70 | 0.55-0.90 |  |
| **25-29 (ref)** |  |  | 0.681 |
| <20 | 1.35 | 1.01-1.80 |  |
| 20-24 | 1.04 | 0.84-1.30 |  |
| 30-34 | 1.05 | 0.85-1.29 |  |
| 35-39 | 1.22 | 0.97-1.55 |  |
| 40+ | 1.20 | 0.79-1.81 |  |
